# Bovine Viral Diarrhea Virus Type 2 Impairs Macrophage Responsiveness to Toll-Like Receptor Ligation with the Exception of Toll-Like Receptor 7

**DOI:** 10.1371/journal.pone.0159491

**Published:** 2016-07-15

**Authors:** Robert G. Schaut, Julia F. Ridpath, Randy E. Sacco

**Affiliations:** 1 Immunobiology Graduate Program, Iowa State University, Ames, Iowa, United States of America; 2 Ruminant Diseases and Immunology Research Unit, National Animal Disease Center, ARS, USDA, Ames, Iowa, United States of America; CEA, FRANCE

## Abstract

*Bovine viral diarrhea virus* (BVDV) is a member of the *Flaviviridae* family. BVDV isolates are classified into two biotypes based on the development of cytopathic (cp) or non-cytopathic (ncp) effects in epithelial cell culture. BVDV isolates are further separated into species, BVDV1 and 2, based on genetic differences. Symptoms of BVDV infection range from subclinical to severe, depending on strain virulence, and may involve multiple organ systems and induction of a generalized immunosuppression. During BVDV-induced immune suppression, macrophages, critical to innate immunity, may have altered pathogen recognition receptor (PRR) signaling, including signaling through toll-like receptors (TLRs). Comparison of BVDV 2 strains with different biotypes and virulence levels is valuable to determining if there are differences in host macrophage cellular responses between viral phenotypes. The current study demonstrates that cytopathic (cp), noncytopathic (ncp), high (hv) or low virulence (lv) BVDV2 infection of bovine monocyte-derived macrophages (MDMΦ) result in differential expression of pro-inflammatory cytokines compared to uninfected MDMΦ. A hallmark of cp BVDV2 infection is IL-6 production. In response to TLR2 or 4 ligation, as might be observed during secondary bacterial infection, cytokine secretion was markedly decreased in BVDV2-infected MDMΦ, compared to non-infected MDMΦ. Macrophages were hyporesponsive to viral TLR3 or TLR8 ligation. However, TLR7 stimulation of BVDV2-infected MDMΦ induced cytokine secretion, unlike results observed for other TLRs. Together, these data suggest that BVDV2 infection modulated mRNA responses and induced a suppression of proinflammatory cytokine protein responses to TLR ligation in MDMΦ with the exception of TLR7 ligation. It is likely that there are distinct differences in TLR pathways modulated following BVDV2 infection, which have implications for macrophage responses to secondary infections.

## Introduction

Bovine viral diarrhea virus (BVDV) is a single stranded, positive sense RNA virus of the *Pestivirus* genus, in the *Flaviviridae* family. BVDV shares many germline similarities to related *Flaviviridae* viruses such as *hepatitis C virus* (HCV) [1]. BVDV can be categorized into two species BVDV1 and BVDV2 as determined by genetic analysis of the 5’ UTR [2]. Symptoms associated with BVDV infection of both species can range from mild to severe fatal acute disease with high mortality rates. Strains of low virulence causing subclinical infection may circulate undetected in a herd for an extended period. Recently, an increased number of severe, acute BVDV field cases have been reported, which in North America, are predominately BVDV2. Infection with viruses isolated from these outbreaks reproducibly resulted in severe disease marked by marked drops in circulating white blood cells, platelets and lymphoid tissues not seen with previously isolated field strains [3]. Therefore, BVDV isolates may be classified as atypical/high virulence (HV) or typical/low virulence (LV), with disease severity correlating to virulence [3]. Cytopathogenicity is an *in vitro* trait of BVDV in which strains belonging to cp biotype induce a cytopathic effect on epithelial cell culture [4]. Cytopathogenicity does not always correlate with disease severity and the majority of BVDV infections involve ncp strains [5]. Isogenic strains of cytopathic and non-cytopathic BVDV isolated from cases of mucosal disease, differ in the cleavage of the viral NS2/3 protein [6]. Evidence suggests that highly virulent ncp strains of BVDV2 induce a cytopathic effect on lymphoid cells that does not involve NS2/3 protein production; therefore via a differing mechanism than cp strains [7]. The two biotypes of BVDV differ in their detection and elimination by the immune system [8]. Few studies have explored immunological differences between BVDV2 HV and LV strains and potential differences in immune modulation are unknown. Specifically, immune responses induced by these various strains of BVDV2 are yet to be fully explored.

BVDV infection may predispose the host to a prolonged susceptibility to secondary bacterial or viral infection, even following recovery. The mechanisms of this immune modulation are unknown to date [9]. In cattle, BVDV is a contributing factor to bovine respiratory disease complex (BRDC), and may predispose to secondary infection by *Mannheimia haemolytica*, *Pasteurella multocida* or involve co-infection with viral agents such as *bovine respiratory syncytial virus* (bRSV), *bovine parainfluenza virus* type 3 (BPIV3) and *bovine herpes virus 1* (BHV-1) [10–13]. Thus, there is a need to understand the underlying mechanisms of disease and immune modulation to better develop anti-viral treatment modalities against BVDV and related *Flaviviridae* members.

Monocytes and tissue resident macrophages (MΦ) are essential cells of the innate immune response to pathogens. MΦ can recognize conserved molecules or components of microorganisms termed pathogen associated molecular patterns (PAMPs) [14]. PAMPs are recognized by pattern recognition receptors (PRRs), which will initiate proinflammatory and anti-viral immune responses. More specifically, Toll-like receptors (TLRs) are a class of PRRs, which are critical to viral and bacterial sensing. As TLRs are conserved over mammalian species, pathogens have developed similar ways to thwart immune recognition by these receptors, [15–17] which in turn lead to a more successful infection with respect to the pathogen.

The initiation of the immune response, after recognition by TLRs, is characterized by cytokine secretion. MΦ are one of the critical cells that secrete IL-1β, TNFα and IL-6. These pro-inflamatory cytokines have been shown to have an anti-viral role [16, 18–20] and subsequently the secretion and pathways involved in induction of these cytokines are targets of pathogens. We found that differentiated MΦ isolated after an *in vivo* BVDV2 infection are impaired in their response to TLR4 ligation [21]; however ligation of other TLRs have yet to be fully explored.

To our knowledge, there have been no published comparative studies examining the effect of BVDV2 strains of varying cytopathogenicity and virulence on MΦ cytokine responses or the effect of these strains on subsequent TLR responsiveness. It was hypothesized that BVDV2 will supress MΦ responses, and secondly BVDV2 strains of varying pathogenicity may induce differential responses in inoculated MΦ cells. BVDV2 infection may impact the ability of MΦ TLRs to appropriately respond to secondary infections. Modulation of responsiveness to viral or bacterial PAMPS could result in enhanced susceptibility to secondary bacterial or viral infection. In this study, we investigated the effects of varying strains of BVDV2 on MDMΦ cytokine expression prior to and following TLR ligation.

## Materials and Methods

### Animals

12 clinically healthy Holsteins of approximately 1–2 years of age were used for blood donors in order to have cells from 9 donor animals per experiment for the four experiments. Animals were negative for BVDV as measured by an immunohistochemistry (IHC) ear notch and RT-PCR of peripheral blood mononuclear cells [22]. Animal procedures employed in these studies were approved by the National Animal Disease Center Institutional Animal Care and Use Committee.

### Monocyte derived macrophage culture

Peripheral blood mononuclear cells were isolated and red blood cells (RBC) lysed with buffered ammonium chloride salt solution [21]. After RBC lysis, cells were washed with sterile phosphate-buffered saline (PBS). Cells were resuspended in complete RPMI 1640 (cRPMI) containing 10% fetal bovine serum (tested commercially and in house to be free of BVDV and antibodies against BVDV), 2 mM L-glutamine, 1% antibiotic–antimycotic solution, and gentamicin sulfate (Life Technologies—Gibco, Carlsbad, CA). Monocytes were isolated by plastic adherence for 2 h as previously described [21]. Ice-cold PBS was added to the plate and adherent cells were removed with a cell scraper. Cells were centrifuged at 1180 x g (Sorvall RC3C Plus, Thermo Scientific, Waltham, MA), and resuspended in cRPMI 1640 at 5x10^6^ cells per mL. Monocytes were plated in 96-well round bottom tissue culture plates in 100 μL medium. Monocytes were cultured for 7 days with media change every 2–3 days to derive MΦ as previously described [21, 23]. Monocytes and subsequently derived macrophages were uniformly positive for CD14 and MHC class II surface expression as determined by flow cytometry (data not shown).

### Viral inoculum and TLR agonists

BVDV strains were cultured as previously described [3, 7, 24]. Briefly, Madin-Darby bovine kidney (MDBK) monolayers were inoculated with the respective viruses when the cells were approximately 70% confluent. After inoculation, cultures were incubated at 37°C for 72–96 hr. Cultures were harvested by freezing at –20°C. After a freeze–thaw cycle, followed by centrifugation for 10 min at 1,000 × *g*, supernatants were collected, aliquoted, and stored at –80°C until use. BVDV2 strains had the following viral titers as determined by histological staining: BVDV2-296c [TCID_50_ 6.8x10^6^], BVDC2-296nc [TCID_50_ 3.8x10^8^], BVDV2-1373 [TCID_50_ 6.22x10^6^], BVDV2-28508-5 [TCID_50_ 2.37x10^6^]. Cell counts were determined using a Coulter Z2 Particle Count and Size Analyzer (BD Biosciences) and inoculated at an MOI of 1. Cells were incubated for 90 minutes with viral inoculum before they were washed twice with 37°C–warmed cRPMI. For studies with TLR agonists, cells were incubated after BVDV2 inoculation for 48 h prior to addition of TLR treatments. TLR agonists were used at the following concentrations: Pam3Cys [5 μg/mL] (Invivogen, San Diego, CA), *Mannheimia haemolytica* LPS [10 μg/mL] (produced in house), *Escherichia coli* LPS [1 μg/mL] (055:B5 Sigma, St. Louis, MO), Poly I:C [50 μg/mL, Sigma], Imiquimod [10 μg/mL] (Invivogen), ssRNA40 LyoVec [10 μg/mL] (Invivogen). Cell viability was measured by acridine orange staining prior to and after virus and TLR treatment and was observed to be >90%. All non-LPS containing reagents were certified free of endotoxin by the manufacturer.

### RNA extraction, cDNA synthesis and qPCR

At 2, 6, 18, and 24 h post BVDV inoculation, cells were lysed with Buffer RLT containing 2-mercaptoethanol (Qiagen, Valencia, CA) and stored at −80°C. Total RNA was isolated using the RNeasy Mini RNA Isolation Kit (Qiagen) and genomic DNA was removed during RNA isolation using an on-column RNase-Free DNase I digestion kit (Qiagen) per manufacturer’s instructions. 500 ng of total RNA from each sample was reverse transcribed using random primers/hexamers and Superscript III (Life Technologies) to generate first strand cDNA. Primers were designed specifically for SYBR Green chemistry with the use of Primer Express v 3.0 (Applied Biosystems, Foster City, CA) or NCBI Primer Blast. Primer annealing temperature was set at 60°C with product size of 100–200 base pairs. Bovine ribosomal protein S9 (RPS9) was used as the endogenous control [25]. Primer set sequences designed for this study are indicated in Table 1 or utilized from previous publications [26, 27]. An Applied Biosystems 7300 Real Time PCR Systems thermocycler was used. Amplification conditions for all genes were the same: 2 min at 50°C, 10 min at 95°C, 40 cycles of 15 s 95°C and 1 min 60°C (measure florescence step) and a dissociation step of 15 s 95°C, 1 min 60°C, 15 s 95°C, 15 s 60°C. Ct values were calculated and normalized to the endogenous control and expressed relative to medium only treatment using the 2^-ΔΔCT^ method [28].

**Table 1:** Primer sequence for bovine targets and control genes.

Target	Sequence (5'-3')	PCR Product Length	Accession no.
IL-1β F	TTC TGT GTG ACG CAC CCG TGC	88	NM_174093.1
IL-1β R	AGC ACA CAT GGG CTA GCC AGC		
TNFα F	CGG GGT AAT CGG CCC CCA GA	281	NM_173966.3
TNFα R	GGC AGC CTT GGC CCC TGA AG		
IL-6 F	TGA GTC TGA AAG CAG CAA GGA	138	NM_173923.2
IL-6 R	TAC TCC AGA AGA CCA GCA GTG G		
IL-8 F	CGC TGG ACA GCA GAG CTC ACA AG	105	NM_173925.2
IL-8 R	GCC AAG AGA GCA ACA GCC AGC T		
IL-12p40 F	TCA AAC CAG ACC CAC CCA AG	201	NM_174356.1
IL-12p40 R	TGT GGC ATG TGA CTT TGG CT		
IL-10 F	TTA CCT GGA GGA GGT GAT G	64	NM_174088.1
IL-10 R	GTT CAC GTG CTC CTT GAT G		
RPS9 F	CGC CTC GAC CAA GAG CTG AAG	66	NM_001101152.2
RPS9 R	CCT CCA GAC CTC ACG TTT GTT CC		

F = forward; R = reverse.

### Searchlight cytokine multiplex assay

50 μL of macrophage (MΦ) supernatants or cytokine standards were incubated for 3 h in duplicate in 96 well, immobilized antibody 4-plex array plates utilizing reagents supplied in the kits (SearchLight, Aushon Biosystems, Billerica, MA). Wells of each plate were commercially coated with antibodies specific for bovine interleukin (IL)-1β, tumor necrosis factor (TNF)-α, and IL-6. The assay was conducted according to manufacturer’s instructions. After chemiluminescent substrate, plates were immediately read on a SearchLight Plate Reader (Aushon Biosystems). The concentrations of cytokines in each sample were determined from standard curves using SearchLight Array Analyst Software v 2.6.2.0 (Aushon Biosystems). The lower limits of detection for the cytokines were: IL-1β 1.9, TNFα 2.4, and IL-6 0.9 pg/mL, respectively.

### Statistical analysis

qPCR cytokine data was analyzed with the outcome variable (2^-ΔΔCT^) log transformed. ΔΔCt values were analyzed using one-way ANOVA (Prism, GraphPad, LaJolla, CA). Bonferroni post test was used to compare replicate means by row to uninfected controls. Cytokine protein data was analyzed using one-way ANOVA (Prism, GraphPad). Results are expressed as means +/- standard errors of the means (SEM).

## Results

### The cytopathic strain BVDV2-296c induced higher levels of proinflammatory cytokine mRNA in bovine MDMΦ than the noncytopathic strain BVDV2-296nc

**Fig 1:**
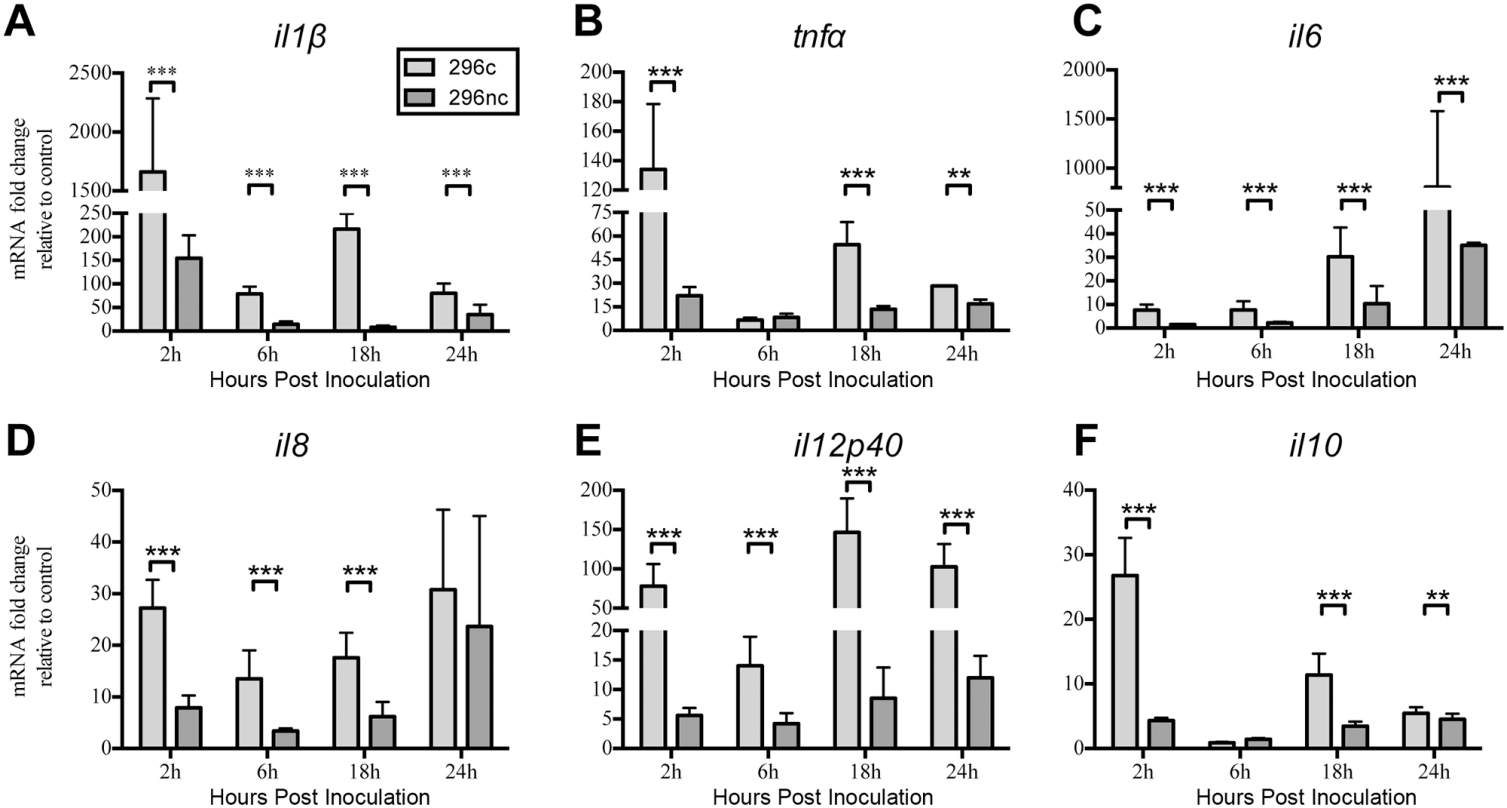
Expression of proinflammatory cytokine gene transcription in MDMΦs inoculated with cytopathic or non-cytopathic BVDV2 strains. MDMΦs were differentiated in 96 well plates for 7 days and inoculated with BVDV2 strains in duplicate at an MOI of 1 with RNA harvested at 2, 6, 18, and 24 h after inoculation. Cytokine mRNA was analyzed by qPCR using ribosomal protein s9 (RPS9) as an endogenous control with fold change expressed relative to uninfected control MDMΦs harvested at the corresponding time points. *il1β* (A), *tnfα* (B), *il6* (C), *il8* (D), *il12p40* (E), *il10* (F) were measured using primer sets specific for bovine genes using SYRB Green chemistry. Bars represent the mean value ± SEM from four different experiments with 9 total donor cattle. *** *P* < 0.001; ** *P* < 0.005.

BVDV2-296c is a cp strain which is genetically identical to BVDV2-296nc except for an inserted sequence in the coding region of the NS2/3 gene of the BVDV2-296c strain [6, 28–32]. The effects of infection with these BVDV strains on MDMΦ cytokine expression have not been examined. Bovine MDMΦs were differentiated for 7 days in 96 well plates and inoculated with an MOI of 1 for each strain and RNA isolated at 2 h, 6 h, 18 h, and 24 h post- inoculation. Cytokines measured displayed similar kinetics throughout the duration of experiments with an initial induction of mRNA followed by another induction of message at 18 h post BVDV2 inoculation with either strain (Fig 1). *il1β* (Fig 1A), *il6* (Fig 1C) and *il12p40* (Fig 1E) were upregulated to a greater extent in MDMΦs inoculated with BVDV2-296c compared to BVDV2-296nc at each time point measured. *tnfα* (Fig 1B) and *il10* (Fig 1F) were induced to a greater extent in BVDV2-296c infected MDMΦs compared to BVDV2-296nc inoculation except for 6 h post treatment. BVDV2-296c inoculated MDMΦs exhibited greater levels of *il8* (Fig 1D) than BVDV2-296nc inoculated MDMΦs except for 24 h post treatment. MDMΦs infected with BVDV2-296c exhibited greatly enhanced levels of proinflammatory cytokines. In contract, BVDV2-296nc inoculation of MDMΦs only modestly induced proinflammatory cytokines and was, in general, significantly (P<0.0001) less than the cytokines induced by BVDV2-296c.

### Infection of MDMΦs with a non-cytopathogenic high virulence BVDV2 strain induced greater proinflammatory cytokine mRNA responses compared to a non cytopathogenic typical/low virulence strain

**Fig 2:**
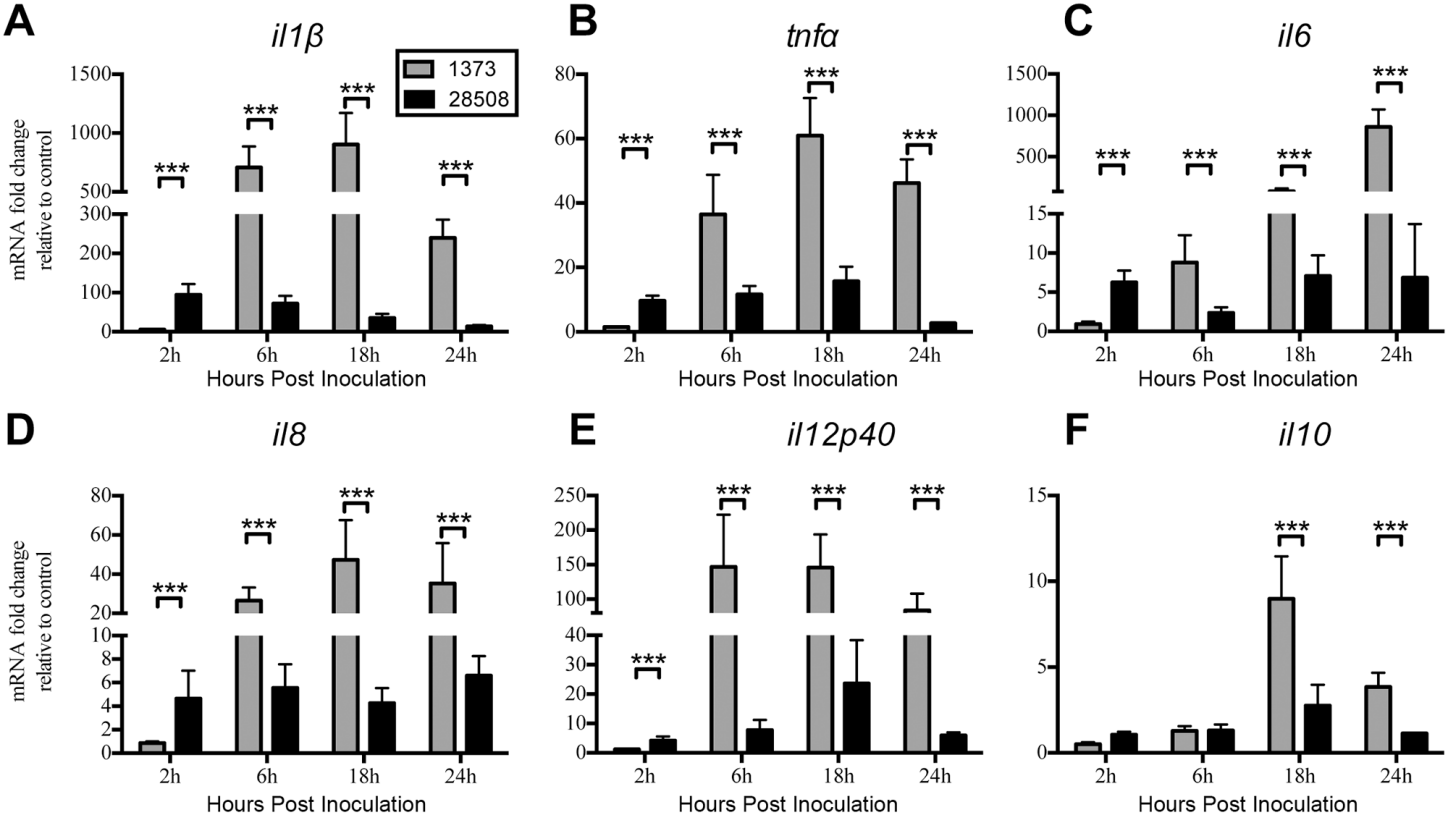
Expression of proinflammatory cytokine gene transcription in MDMΦs inoculated with high and low virulence BVDV2 strains. MDMΦs were differentiated in 96 well plates for 7 days and inoculated with BVDV2 strains in duplicate at an MOI of 1 with RNA harvested at 2, 6, 18, and 24 h after inoculation. Cytokine mRNA was analyzed by qPCR using ribosomal protein s9 (RPS9) as an endogenous control with fold change expressed relative to uninfected control MDMΦs harvested at the corresponding time points. *il1β* (A), *tnfα* (B), *il6* (C), *il8* (D), *il12p40* (E), *il10* (F) were measured using primer sets specific for bovine genes using SYRB Green chemistry. Bars represent the mean value ± SEM from four different experiments from 9 total donor cattle. *** P < 0.001.

BVDV2 strains demonstrate vast differences in virulence *in vivo* and can be classified as either high/atypical or low/typical virulence strains [3]. BVDV2 strain BVDV2-1373 is a high virulence (HV) strain which causes severe acute disease in cattle; whereas strain BVDV2-28508-5 is a low/typical virulence (LV) BVDV2 strain which causes mild to no clinical disease. Differences between virulence strains and the effects on cytokine expression in macrophages have not been explored *in vitro*, and we hypothesized there would be an increase in inflammatory cytokines associated with higher virulent BVDV strains. In agreement with our hypothesis, we found that HV BVDV2-1373 induced greatly enhanced proinflammatory cytokine expression in inoculated cells compared to LV BVDV2-28508-5 (Fig 2). However, HV BVDV2-1373 induced an initial mRNA expression of cytokines in MDMΦs at 6h post inoculation as opposed to the other BVDV2 strains in which cytokine expression was beginning to be induced as early as 2 h post inoculation (Figs 1 and 2). *il1β* (Fig 2A), *tnfα* (Fig 2B), *il6* (Fig 2C), *il8* (Fig 2D), and *il12p40* (Fig 2E) were enhanced to a greater extent in MDMΦs infected with HV BVDV2-1373 at 6, 18, 24 h post inoculation compared to LV BVDV2-28508 infected MDMΦs. Additionally, *il10* (Fig 2F) was increased in MDMΦs inoculated with HV BVDV2-1373 at 18 and 24 h post inoculation compared to LV BVDV2-28508 inoculated cells. Overall, HV BVDV2-1373 infected MDMΦs demonstrated a greater level of proinflammatory cytokine expression starting at 6h post infection compared to LV BVDV2-28508 infected cells. It is of note that HV BVDV2-1373 did not induce a proinflamatory cytokine response at 2 h post inoculation compared to the other three strains of BVDV2 used in our experiments.

### Cytopathic strain BVDV2-296c induced IL-6 secretion in infected MDMΦs, whereas all other strains did not induce secretion of proinflammatory cytokines

**Fig 3:**
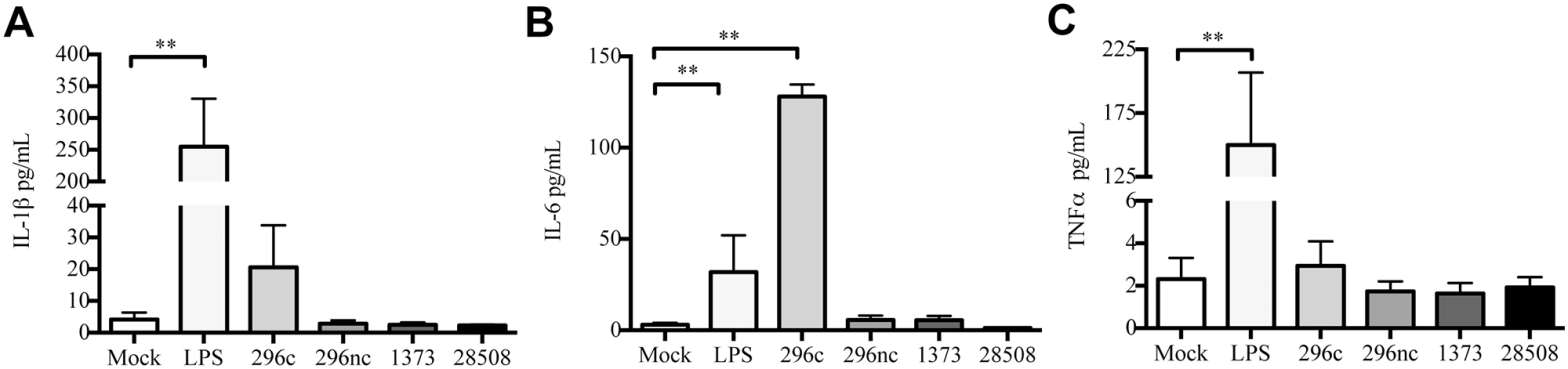
Proinflammatory cytokine secretion of BVDV2 inoculated or LPS stimulated MDMΦs 24 h after treatment. MDMΦs were differentiated in 96 well plates for 7 days and inoculated with BVDV2 strains in duplicate at an MOI of 1 or 2 μg/mL LPS with cell supernatants harvested at 24 h after treatment. Cytokine protein was analyzed by Searchlight Array using analytes specific for bovine IL-1β (A), IL-6 (B) and TNFα (C) with 50 μL of cell supernatant analyzed in duplicate. Cytokines were quantified by generation of standard curves against recombinant bovine cytokines provided by the manufacturer of the Searchlight platform. Bars represent the mean value ± SEM from four different experiments from 9 total donor cattle. ** P < 0.001.

Many studies of BVDV focus on cytokine mRNA expression in response to viral inoculation *in vitro*. There have been no comparative studies of BVDV2 strains *in vitro* in which MDMΦ cytokine secretion is explored. As we observed an induction in cytokine mRNA, we investigated the protein secretion of MDMΦs in response to BVDV2-296c, BVDV2-296nc, BVDV2-1373 and BVDV2-28508. Interestingly, each of the viral strains did not induce cytokine secretion, except for BVDV2-296c (Fig 3). LPS was used as a positive control in these experiments. IL-1β (Fig 3A), and TNFα (Fig 3C) secretion was not increased in the viral treated groups compared to control. Interestingly, IL-6 (Fig 3B) was increased only in the BVDV2-296c-treated MDMΦs compared to both LPS stimulated cells or cells inoculated with other virus strains. These results suggest that there is a disconnect between mRNA expression and cytokine secretion in BVDV2 inoculated MDMΦs. It is noteworthy that although BVDV2-296c did induce IL-6 secretion, which mirrors gene transcription, IL-1β and TNFα were not induced. It is possible that the cytopathic nature of BVDV2-296c may be a factor in the induction of IL-6. Additionally, examination of later time points of 48 h and 72 h post inoculation did not yield any differences in expression of secreted cytokine protein (S1 Fig).

### BVDV2 infected MDMΦs stimulated with bacterial TLR agonists exhibited reduced proinflammatory cytokine secretions

**Fig 4:**
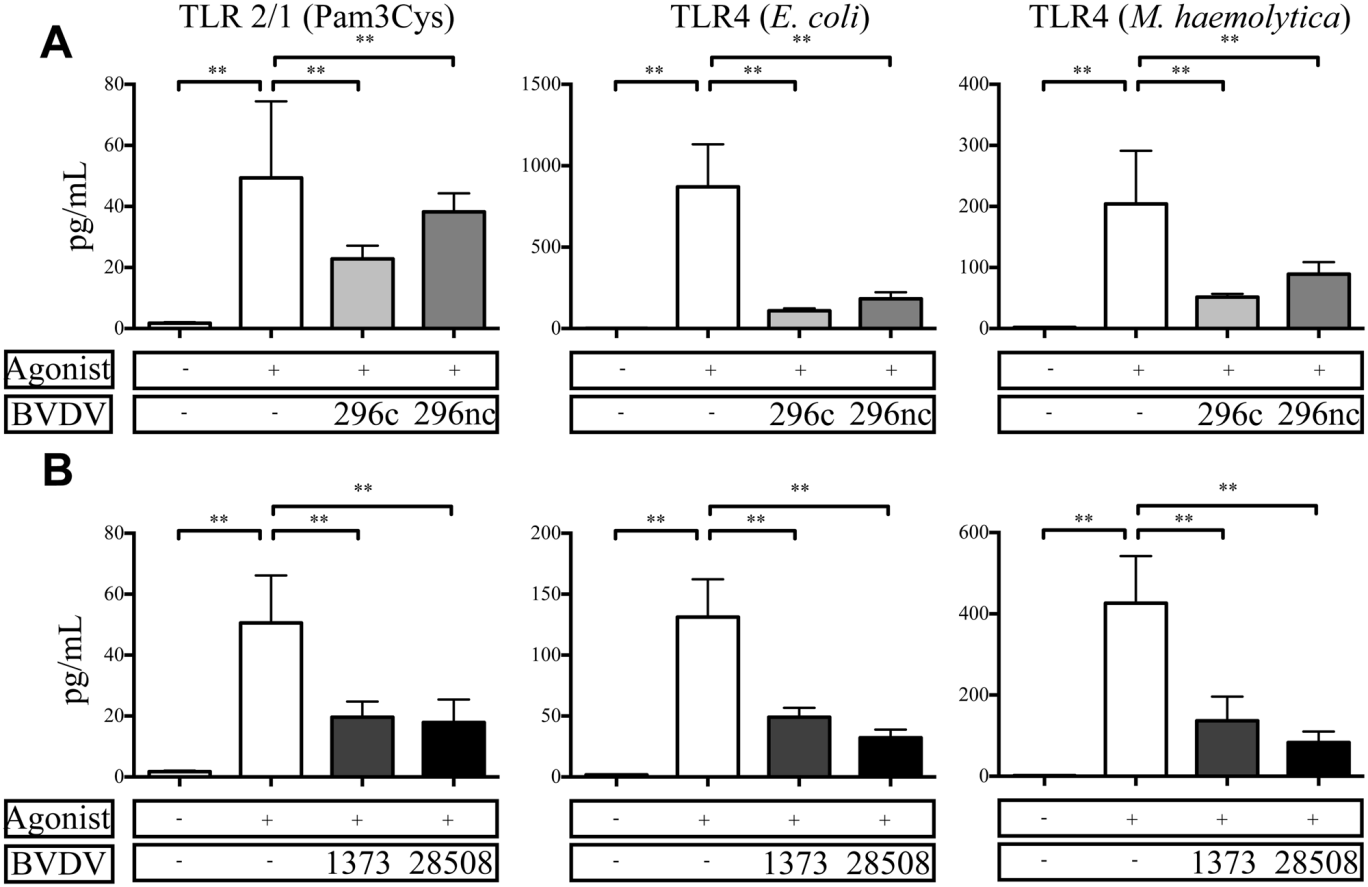
TNFα protein secretion from BVDV2 infected monocyte derived macrophages after stimulation with bacterial TLR agonists. MDMΦs were differentiated in 96 well plates for 7 days and inoculated with BVDV2 strains with an MOI of 1 for 48 h prior to stimulation with Pam3Cys [5 μg/mL], *M*. *haemolytica* LPS [10 μg/mL], or *E*. *coli* (055:B5) LPS [1 μg/mL]. Cell supernatants were analyzed for TNFα protein concentration 24 h after TLR stimulation and measured by Searchlight Array platform. Data from infection with cytopathic or noncytopathic strains are in the top panel (A) and from high or low virulence strains indicated in the lower panel (B). Bars represent the mean value ± SEM from four different experiments from 9 total donor cattle. ** P < 0.001 compared to uninfected, TLR stimulated cells.

As the majority of BVDV2 strains alone did not induce proinflammatory cytokine secretion, this interesting observation lead us to explore whether BVDV2 would inhibit cytokine secretion in response to bacterial TLRs. Few studies have investigated *E*. *coli* LPS responsiveness in BVDV1 inoculated MΦ derived *in vitro* [33–36] and there have been no studies comparing varying strain of BVDV2 to multiple types of bacterial TLRs. TNFα protein secretion was decreased in virus inoculated MDMΦs in response to TLR stimulation compared to TLR-stimulated uninfected controls (Fig 4). For MDMΦs inoculated with BVDV2-296c or BVDV2-296nc, there were no dramatically different levels of suppression between the treatment groups (Fig 4A). Noteworthy is that both *E*. *coli* and *M*. *haemolytica* LPS stimulation of MDMΦs inoculated with BVDV2-296c or BVDV2-296nc exhibited less TNFα protein secretion than those stimulated with Pam3Cys. Similarly, MDMΦs inoculated with BVDV2-1373 or BVDV2-28508, there were no statistically different levels of suppression between the inoculated cells (Fig 4B). Interestingly, *M*. *haemolytica* LPS stimulation was not as suppressed in BVDV2 inoculated BVDV2-1373 or BVDV2-28508 MDMΦs as with the BVDV2-296c or BVDV2-296nc inoculated cells. In general, virulence and cytopathogenicity do not necessarily impact the level of suppression in BVDV2 infected MDMΦs stimulated with bacterial TLRs, as all groups were diminished in their cytokine response to bacterial TLR ligation. Furthermore, levels of secreted IL-1β and IL-6 were similarly reduced in BVDV2 inoculated and TLR-stimulated cells (S2 and S3 Figs).

### BVDV2 infected MDMΦs respond to TLR7 ligation; however other viral TLR agonists failed to induce similar cytokine responses

**Fig 5:**
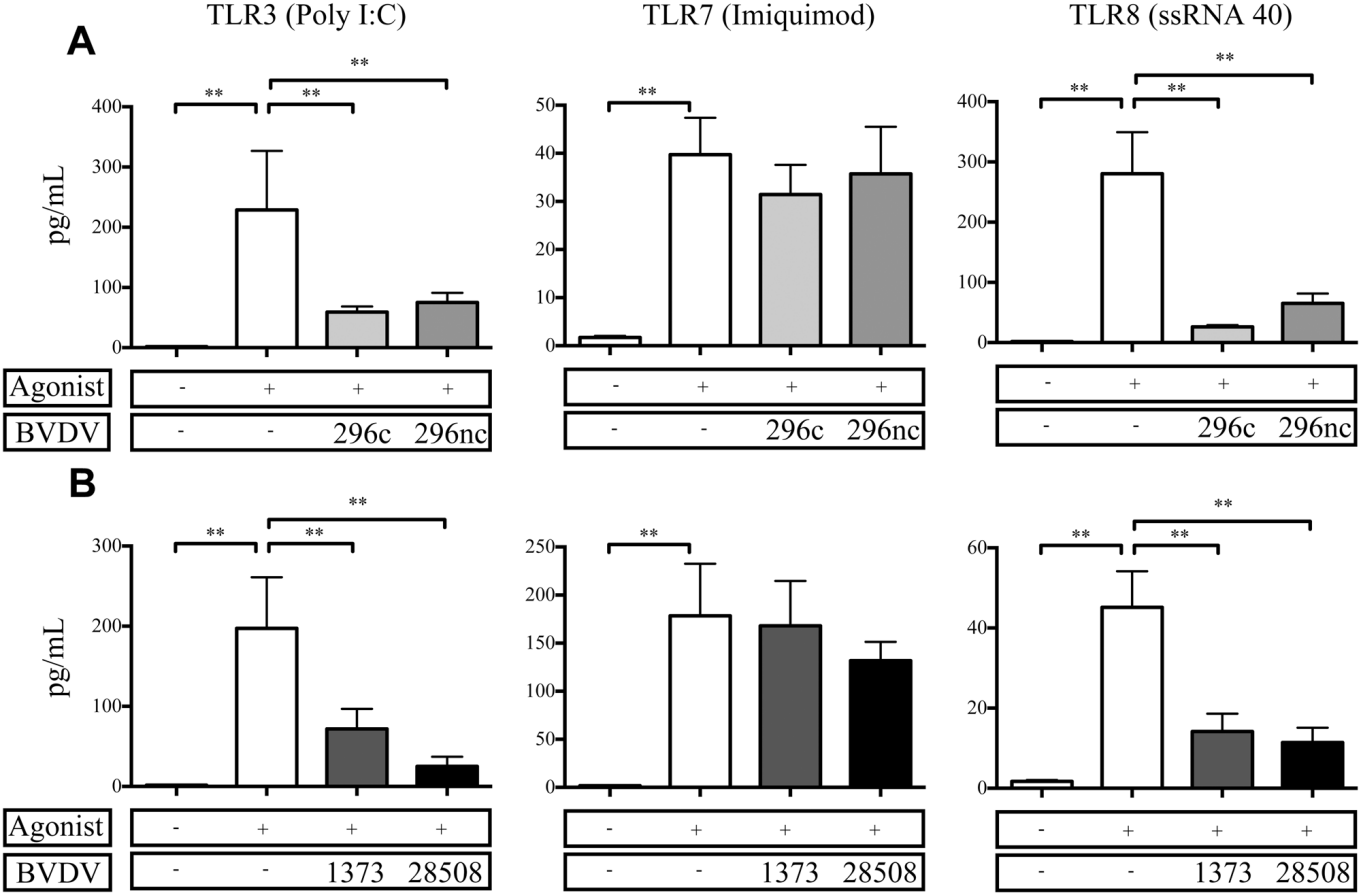
TNFα protein secretion from BVDV2 infected MDMΦ after stimulation with viral TLR agonists. MDMΦs were differentiated in 96 well plates for 7 days and inoculated with BVDV2 strains with an MOI of 1 for 48 h prior to stimulation with Poly I:C [50 μg/mL], Imiquimod [10 μg/mL], ssRNA40 LyoVec [10 μg/mL]. Cell supernatants were analyzed for TNFα protein concentration 24 h after TLR stimulation and measured by Searchlight Array platform. Incubation with cytopathic and noncytopathic strains are in the top panel (A) or high and low virulence strains indicated in the lower panel (B). Bars represent the mean value ± SEM from four different experiments from 9 total donor cattle. ** P < 0.001 compared to uninfected, TLR stimulated cells.

As we observed a decrease in cytokine protein expression from BVDV2 inoculated MDMΦs in response to bacterial TLRs, we decided to explore TLRs specific to viral PAMPs. BVDV infection *in vivo* may predispose an animal to secondary viral infection, and a deficit in viral TLR responsiveness could potentially contribute to this observation. Previous comparative studies of BVDV2 strains and the effects of various viral TLRs *in vitro* have been limited. Interestingly, TNFα protein was not suppressed in BVDV2 inoculated MDMΦs in response to TLR7; however responses to both TLR3 and TLR8 were suppressed (Fig 5). There were no statistically different levels of suppression for TNFα expression in either cytopathic BVDV2-296c or non-cytopathic BVDV2-296nc inoculated MDMΦs (Fig 5A). Similarly, there were no differences in the amount of suppression of TNFα protein secretion between the MDMΦs infected with either HV BVDV2-1373 or LV BVDV2-28508 (Fig 5B) and stimulated with TLR agonists; however TLR7 agonists were able to induce a response in each of the virus infected treatment groups, with each of them statistically similar to agonist stimulation alone. Similar to findings with bacterial TLR agonists, cytopathogenicity or virulence does not effect the level of suppression for BVDV2 infected MDMΦs stimulated with viral TLR agonists and in which TLR3 and TLR8 were both diminished in their ability to respond in virally inoculated cells. Likewise, IL-1β and IL-6 from virally inoculated cells stimulated with TLR3 and TL8 agonists demonstrated a reduction in cytokine secretion (S2 and S3 Figs).

## Discussion

This study demonstrated that BVDV2 infection of MDMΦ induces robust proinflammatory cytokine mRNA responses, which differ dependent on the cytopathogenicity and virulence of the viral strains. Nevertheless, there was no enhancement of TNFα or IL-1β protein secretion, whereas IL-6 was only detected in response to the cp strain utilized in this study. Future experiments should examine the mechanism whereby BVDV2 infection inhibits secretion of specific cytokines. It is noteworthy that the response to secondary bacterial or viral TLR stimuli after BVDV2 infection was generally reduced compared to uninfected cells. However, TLR7 signaling after BVDV infection was not abrogated.

BVDV1 has been demonstrated to decrease: lymphocyte proliferation [36], chemotaxis in monocytes [37, 38], microbicidal activity of neutrophils [39, 40], TLR receptors [29] and expression of complement, Fc receptors and chemokine production of alveolar macrophages [41] which taken together suggests a broad immune modulation within an infected host. It has been suggested that monocytes or macrophage cells are critical in BVDV infection for disseminating virus [42, 43] and initiating apoptosis of T cells during infection [44, 45]. Severe lymphopenia is seen in infected animals, however monocytes are not reduced in the periphery [3, 46, 47]. This observation suggests that monocytes or macrophages may play a critical role in recovery from BVDV infection, as they are not depleted throughout the course of infection.

This study compared differences between BVDV2 isogenic strains or strains of varying virulence and the impact on infected MDMΦ cytokine mRNA responses. In the experimental design of these experiments, MDMΦ were inoculated with BVDV2 strains, followed 90 min later by removal of the inoculum, washing and addition of fresh medium. Therefore; any non-specific activating factors would likley not illicit a measurable response in the inoculated MDMΦ, which have been shown return to a quiescent state rapidly after removal of these non-specific activating factors [48]. It is noteworthy that we saw a robust cytokine mRNA response, which was more pronounced in the cp and hv BVDV2 strains (Figs 1 and 2). Of note, the work of others have demonstrated that *tnfα* gene transcription is enhanced in PBMCs infected with cp BVDV1 strains, however comparison to non-cytopathic strains was not examined [49]. Our findings suggest only an increase in expression of mRNA, not protein secretion, from infected MDMΦ. It is possible that BVDV2 can impact TNFα protein expression by modification to the 3’ UTR of the *tnfα* mRNA sequence eliciting it to degrade posttranscriptionally as observed in natural control of inflammatory processes by the cell [50]. The cytopathic BVDV2-296c strain did induce IL-6 secretion in MDMΦ, which can help to induce apoptosis under certain conditions [51], therefore the induction of this cytokine may be criticial to cp responses of MDMΦ.

We demonstrated a disconnect between mRNA expression and cytokine protein secretion in each of the BVDV2 inoculated MDMΦ. Previous studies have demonstrated a disassociation between mRNA expression and protein production [52–54]. Differences between mRNA expression and protein production may be related to cellular physiological functions, such as post-translational modifications that inhibit protein production [55, 56], sequestering of protein which blocks secretion [57] or production of inactive forms of cytokines which require secondary mechanisms for activation and secretion [58]. Nonetheless, it should be noted that the dissociation between cytokine mRNA expression and protein secretion may be a trait of BVDV2 pathogenesis.

Similar to our finding, ncp BVDV1 strains have been shown to decrease intracellular protein expression of IL-1β and TNFα [26]. It is of note, another *Flaviviridae* family member, dengue virus, demonstrated reduced expression of proinflammatory cytokines IL-1β, IL-8 and TNFα in whole blood from infected children; however this decrease in expression correlated to heightened clinical disease severity [59]. Overall, our research and the findings of others suggest that BVDV reduces or inhibits cytokine protein secretion from infected cells.

Noteworthy is the observation that hv BVDV2 strain BVDV2-1373 induced proinflammatory cytokine gene transcription begining at 6 h post inoculation compared to the MDMΦ response to the other strains which occurred beginning at 2 h post inoculation. This observation may indicate that this particular strain of BVDV2 is unique compared to the other strains we studied, as it is either not recognized as early by the MDMΦ to induce cytokine mRNA at 2 h or is recognized by differing innate mechanisms.

As far as we are aware, this is the only published study to date in which cytokine responses of BVDV2-infected MDMΦ to multiple TLR agonists has been explored. However, a previous study demonstrated that bone marrow derived macrophages (BMMΦ) infected with BVDV1 *in vitro* have been shown to secrete less TNFα protein in response to LPS or bacterial infection compared to uninfected BMMΦs [36]. Similarly, TLR4 hyporesponsiveness has been demonstrated in MDMΦ from chronically infected HCV patients whereas this observation correlated to more severe disease and worse clinical outcome [60, 61]. Likewise, *Flaviviral* infections including *yellow fever virus* (YFV) or *St*. *Louis encephalitis virus* (SLEV), reduced IL-1β levels in LPS-stimulated MDMΦ compared to uninfected control cells [62]. Similar to these findings, we demonstrated that varying TLR4 agonists as well as TLR2/1 agonists are diminished in their ability to promote pro-inflammatory cytokine secretion in MDMΦ after BVDV2 infection. Interestingly, each biotype suppressed MDMΦ cytokine secretion in response to bacterial TLR stimulation.

As with bacterial TLR ligation, there is evidence that BVDV infection can decrease responsiveness to a double-stranded (ds)-RNA viral recognition receptor, TLR3 [26, 63]. The dampened TLR3 response can be attributed to the BVDV ubiquination and degradation of interferon regulatory factor (IRF)-3 directly downstream of TLR3 activation by means of interaction with Npro protein of BVDV [64–66]. Thus, we demonstrated a reduction in viral TLR responsiveness in MDMΦ evidenced by a reduction in cytokine secretion by both TLR3 and TLR8 stimulation after BVDV2 infection (Fig 5). Intriguingly, TLR7 ligation was not dampened after BVDV2 infection and was the only TLR tested that was statistically similar or enhanced in cytokine secretion compared to uninfected control cells. These findings along with the data from this study of bacterial and viral TLR ligation (Figs 4 and 5), demonstrate that there is a TLR hyporesponsivness induced by *Flaviviridae*, including BVDV infection.

It was previously shown that there were no significant upregulation in TLR gene transcription after BVDV1 inoculation of MDMΦ cells [67]. In agreement with these findings, we did not observe differences in TLR gene expression in MDMΦ infected with several strains of BVDV2 (data not shown). In contrast, others have demonstrated differences in type-1 interferon responses in monocyte and monocyte derived dendritic cells infected with two ncp BVDV strains, with a suggested role for TLR7 [68]. Furthermore, microarray analysis which compared BVDV1-ncp to BVDV1-cp strains did find evidence of differential expression of TLR 2, TLR 3, TLR4, and TLR7 genes [69]. Interestingly the authors also reported that BCL2 was increased in the BVDV1-cp inoculated MDMΦ. BCL2 is a molecule important to receptor-activated JNK signaling and cell survival [70]. In that regard, viral modulation of TLR receptor expression and associated signaling complexes would impact cytokine responses to subsequent ligation.

Taken together, the findings in this study demonstrate that the BVDV2 strains vary in their induction of cytokine mRNA responses from MDMΦ. Although a robust mRNA response was observed, protein secretion was not. In addition, subsequent TLR stimulation of BVDV2-infected MDMΦ resulted in a hyporesponsive cytokine response compared to uninfected MDMΦ. However, TLR7 ligation induced cytokine responses in infected MDMΦ. Interestingly, MDMΦ infected with isogenic strains of cp/ncp BVDV2 did not differ in suppression of TLR stimulated cytokine responses, indicating that NS2, NS3 or NS2/3 may not be an explanation of this observation; however IL-6 secretion may be mediated by NS2 or NS3. These results demonstrate that BVDV2 infection generally blocks TLR responsiveness and provide information on how this virus may contribute to susceptibility to secondary infection.

## Supporting Information

S1 Fig IL-1β and IL-6 and protein expression of MDMΦ infected with BVDV2 strains at 48 and 72 h post infection. MDMΦs were differentiated in 96 well plates for 7 days and inoculated with BVDV2 strains with an MOI of 1. Cell supernatants were analyzed for protein concentration 48 h (A) or 72 h (B) after infection and measured by Searchlight Array platform. Bars represent the mean value ± SEM from four different experiments from 9 total donors. ND = no cytokine detected. ** P < 0.001, * P < 0.05 compared to uninfected, TLR stimulated cells.
(TIF)

S2 Fig IL-1βand IL-6 protein expression in response to TLR stimulation in MDMΦ infected with BVDV2 strains 296c or 296nc. MDMΦs were differentiated in 96 well plates for 7 days and inoculated with BVDV2 strains with an MOI of 1 for 48 h prior to stimulation with Pam3Cys [5 μg/mL], *M*. *haemolytica* LPS [10 μg/mL], *E*. *coli* (055:B5) LPS [1μg/mL], Poly I:C [50μg/mL], Imiquimod [10μg/mL], or ssRNA40 LyoVec [10μg/mL]. Cell supernatants were analyzed for protein concentration 24 h after TLR stimulation and measured by Searchlight Array platform. Addition of agonist or cytopathic or noncytopathic strains are indicated below each bar graph. Bars represent the mean value ± SEM from four different experiments from 9 total donors. ** P < 0.001, * P < 0.05 compared to uninfected, TLR stimulated cells.
(TIF)

S3 Fig IL-1βand IL-6 protein expression in response to TLR stimulation in MDMΦ infected with BVDV2 strains 1373 or 28508. MDMΦs were differentiated in 96 well plates for 7 days and inoculated with BVDV2 strains with an MOI of 1 for 48 h prior to stimulation with Pam3Cys [5 μg/mL], *M*. *haemolytica* LPS [10 μg/mL], *E*. *coli* (055:B5) LPS [1 μg/mL], Poly I:C [50 μg/mL], Imiquimod [10μg/mL], or ssRNA40 LyoVec [10 μg/mL]. Cell supernatants were analyzed for protein concentration 24 h after TLR stimulation and measured by Searchlight Array platform. Addition of agonist or strains hv1373 or lv28508 are indicated below each bar graph. Bars represent the mean value ± SEM from four different experiments from 9 total donors. ** P < 0.001, * P < 0.05 compared to uninfected, TLR stimulated cells.
(TIF)
