# Supplementary figures and images for: Bovine Viral Diarrhea Virus Type 2 Impairs Macrophage Responsiveness to Toll-Like Receptor Ligation with the Exception of Toll-Like Receptor 7
Source: PLoS One. 2016 Jul 15;11(7):e0159491. doi: 10.1371/journal.pone.0159491 (PMC4946783; doi:10.1371/journal.pone.0159491)

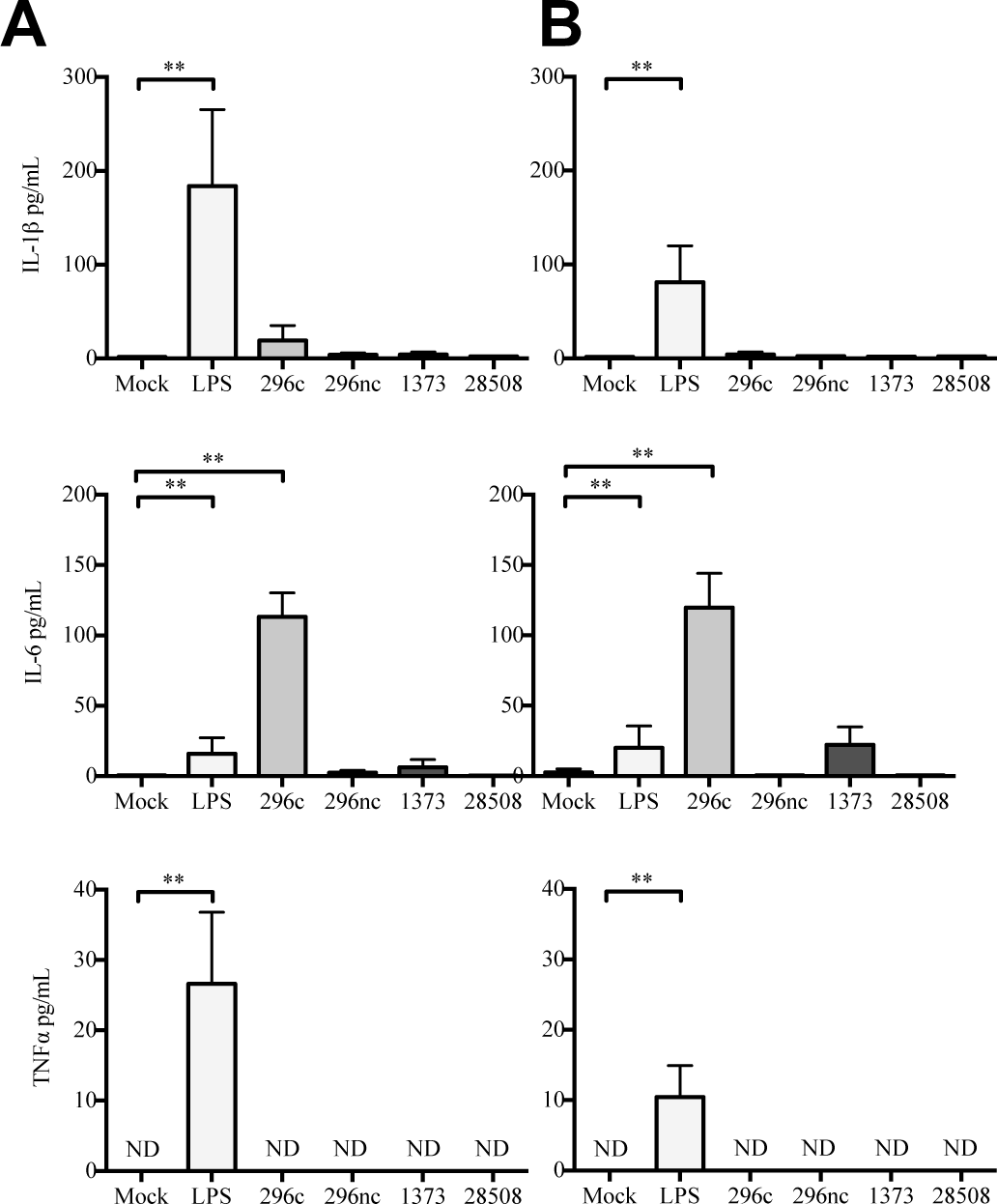

Supplement: S1 Fig — MDMΦs were differentiated in 96 well plates for 7 days and inoculated with BVDV2 strains with an MOI of 1. Cell supernatants were analyzed for protein concentration 48 h (A) or 72 h (B) after infection and measured by Searchlight Array platform. Bars represent the mean value ± SEM from four different experiments from 9 total donors. ND = no cytokine detected. ** P < 0.001, * P < 0.05 compared to uninfected, TLR stimulated cells. (TIF) [file pone.0159491.s001.tif]

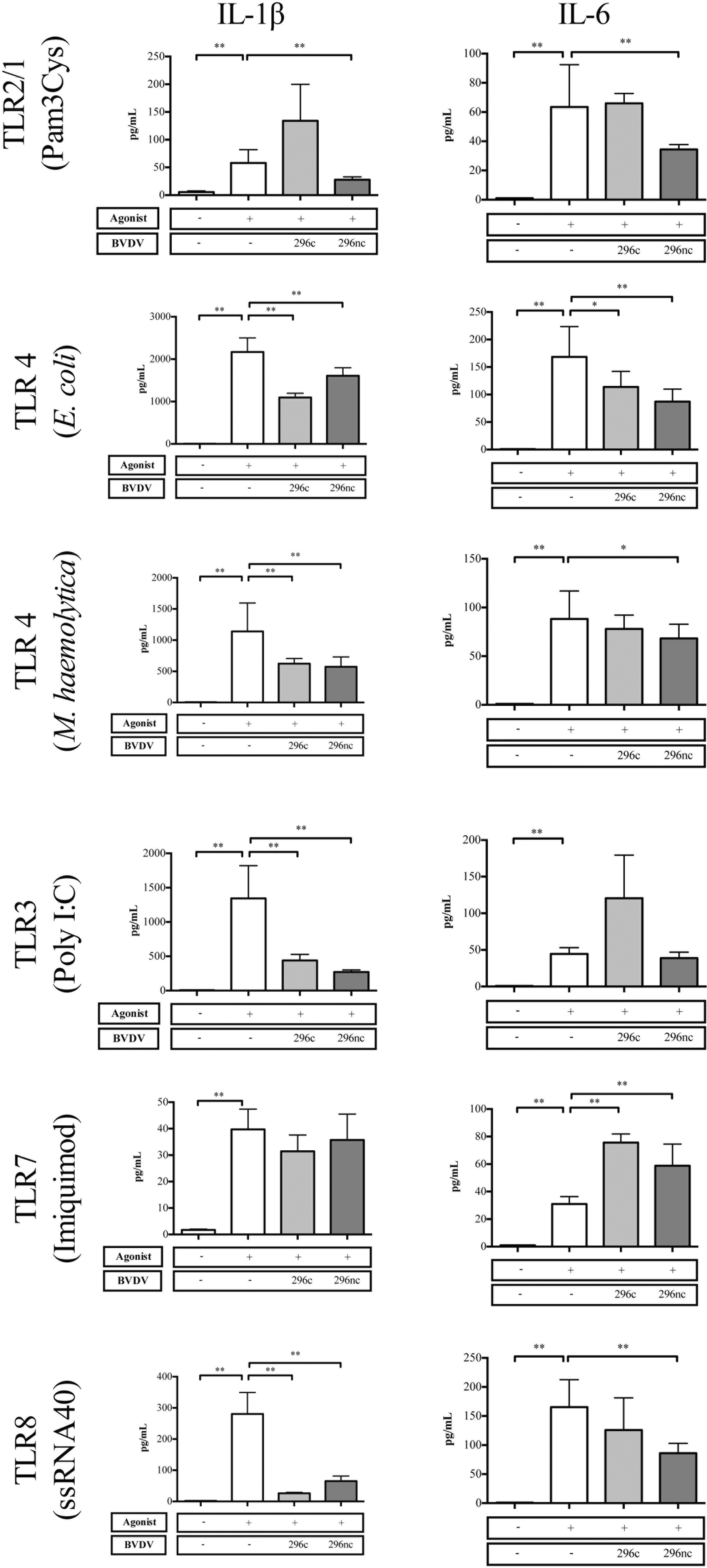

Supplement: S2 Fig — MDMΦs were differentiated in 96 well plates for 7 days and inoculated with BVDV2 strains with an MOI of 1 for 48 h prior to stimulation with Pam3Cys [5 μg/mL], M. haemolytica LPS [10 μg/mL], E. coli (055:B5) LPS [1μg/mL], Poly I:C [50μg/mL], Imiquimod [10μg/mL], or ssRNA40 LyoVec [10μg/mL]. Cell supernatants were analyzed for protein concentration 24 h after TLR stimulation and measured by Searchlight Array platform. Addition of agonist or cytopathic or noncytopathic strains are indicated below each bar graph. Bars represent the mean value ± SEM from four different experiments from 9 total donors. ** P < 0.001, * P < 0.05 compared to uninfected, TLR stimulated cells. (TIF) [file pone.0159491.s002.tif]

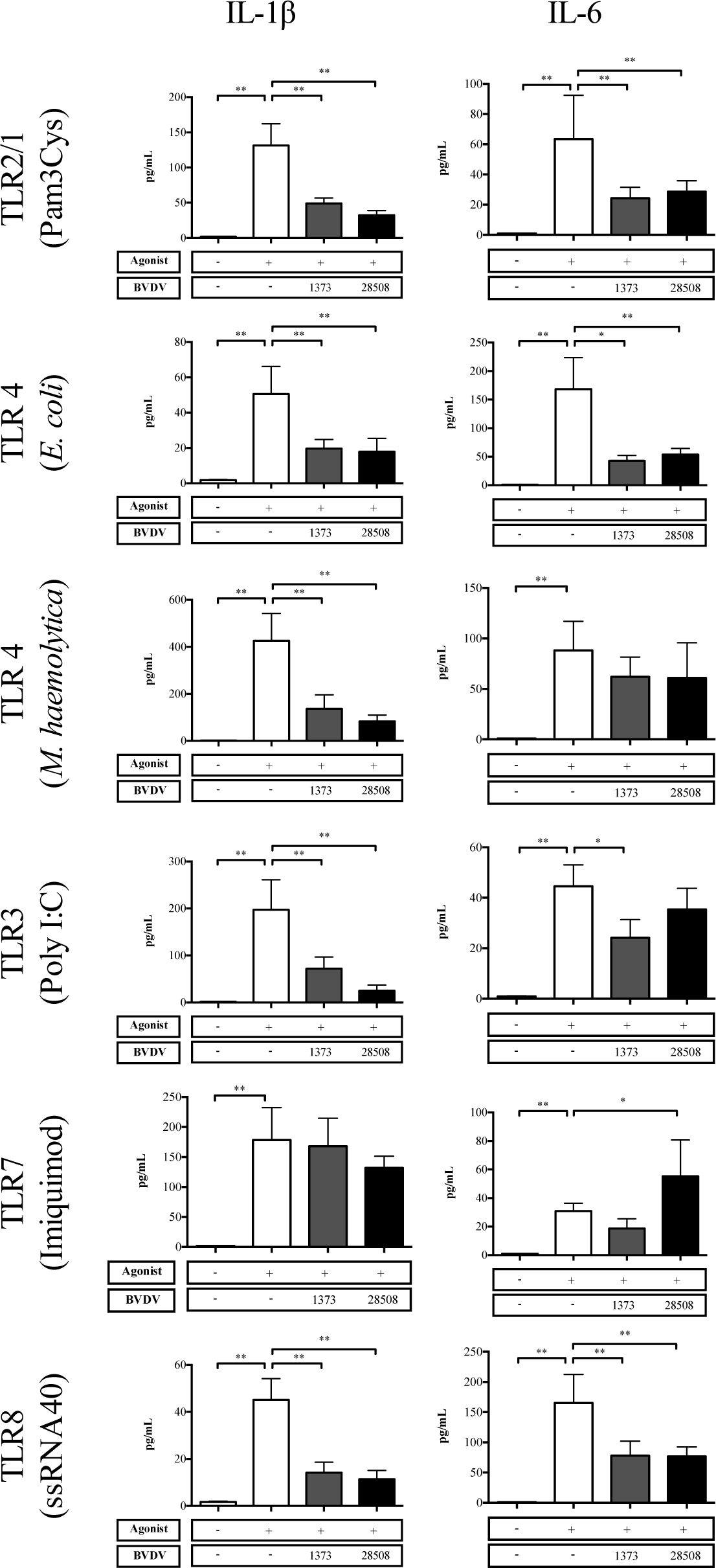

Supplement: S3 Fig — MDMΦs were differentiated in 96 well plates for 7 days and inoculated with BVDV2 strains with an MOI of 1 for 48 h prior to stimulation with Pam3Cys [5 μg/mL], M. haemolytica LPS [10 μg/mL], E. coli (055:B5) LPS [1 μg/mL], Poly I:C [50 μg/mL], Imiquimod [10μg/mL], or ssRNA40 LyoVec [10 μg/mL]. Cell supernatants were analyzed for protein concentration 24 h after TLR stimulation and measured by Searchlight Array platform. Addition of agonist or strains hv1373 or lv28508 are indicated below each bar graph. Bars represent the mean value ± SEM from four different experiments from 9 total donors. ** P < 0.001, * P < 0.05 compared to uninfected, TLR stimulated cells. (TIF) [file pone.0159491.s003.tif]
